# Unidentified Branches of the Posterior Femoral Cutaneous Nerve and Persistent Neuropathy

**DOI:** 10.7759/cureus.29447

**Published:** 2022-09-22

**Authors:** Michelle R Jennette, David Bailey, Neel Patel, Elias Rizk

**Affiliations:** 1 Department of Cellular and Molecular Physiology, Penn State College of Medicine, Hershey, USA; 2 Neurosurgery, Penn State Health Milton S. Hershey Medical Center, Hershey, USA; 3 Neurological Surgery, Penn State Health Milton S. Hershey Medical Center, Hershey, USA

**Keywords:** pudendal neuralgia, inferior clunial neuralgia, posterior femoral cutaneous nerve neuropathy, gluteal neuropathy, gluteal surgery

## Abstract

The posterior femoral cutaneous nerve (PFCN) is an extensive nerve with numerous collateral branches which provide cutaneous innervation to 2/3^rds^ of the posterior thigh, the infragluteal fold, as well as the lateral anal region, scrotum, and labia majora through its inferior cluneal and pudendal nerve branches. It has been noted in multiple studies that patients can experience persistent PFCN neuropathy after surgery for decompression of known collateral branches. In this study, we used 17 formaldehyde (7 male and 10 female) perfused cadavers obtained from Hershey Medical Center’s donor program to study the branching patterns of the PFCN. As a result, we found that 41% of individuals have an unidentified proximal branch of PFCN that recurs over the inferolateral border of the gluteus maximus, suggesting other areas of potential compression or nerve entrapment that could lead to persistent PFCN neuropathy that’s not improved after treatment for sciatic, pudendal, or inferior cluneal neuralgia. We hope these findings allow clinicians to modify current surgical techniques and improve patients’ post-operative quality of life.

## Introduction

The posterior femoral cutaneous nerve (PFCN) is a long cutaneous nerve that exits the pelvis through the sciatic foramen and courses inferiorly down the posterior thigh [1,2]. With many terminal branches, the PFCN provides extensive sensation to multiple regions, including 2/3rds of the posterior thigh, the entire infragluteal fold, and small portions of the lateral anal/perineal region [1,3]. Considering its widespread innervation and many collateral branches, PFCN neuropathy has been commonly reported, primarily due to iatrogenic etiology. While idiopathic neuropathy of the PFCN has been described [4], iatrogenic neuropathy to the PFCN has been recorded after hip replacements, sacral reconstructions, gluteal injections for posterior thigh flap harvesting, and proximal hamstring repair [5-11]. PFCN neuropathy further leads to varying dysesthesias, pain, numbness, paresthesia, and cluneal neuralgia [3,10,12,13]. Therefore, knowledge of its anatomical course and terminal branching patterns is essential to avoid iatrogenic injury during gluteal surgeries. In this study, we detail the branching pattern of the PFCN as it emerges at the inferior edge of the gluteus maximus muscle to determine any unidentified branches that may result in PFCN neuropathy. Our overall objective of this study is to identify a potential cause of persistent PFCN neuropathy in the hope that it will help reduce iatrogenic PFCN injury and improve patient care.

## Materials and methods

Dissections

For this study, 14 unilateral and 3 bilateral dissections were performed on 17 formaldehyde-perfused cadavers. Due to the number of cadavers and resources available, only three bilateral dissections were performed. The PFCN was located via a parasagittal, vertical incision on the posterior thigh from the mid-gluteal region to the popliteal fossa for all dissections. If the most inferior terminal branches of PFCN were not visible, the incision was extended to the most superior portion of the gastrocnemius muscle. This vertical incision was made in a parasagittal nature closer to the medial aspect of the thigh to avoid transecting the PFCN or any of its branches. Similar techniques were followed when removing the fascia lata over the PFCN and surrounding musculature. The PFCN was located just deep to the fascia lata, coursing inferiorly on the biceps femoris muscle. A full neurolysis technique was performed using blunt dissection, completely freeing the PFCN from all surrounding connective tissue until the parent and terminal branches were visible. If the PFCN could not be visualized due to stiffness from preservation, 4.5"-7" retractors were used to retract the skin, adipose tissue, and fascia lata. The use of retractors and large incisions were used to visualize the variation in PFCN branching patterns solely and would not be used under normal surgical conditions.

Identifying terminal branching patterns

After the PFCN had been successfully identified, all terminal branches were liberated from surrounding tissues and documented. Due to the possibility of the PFCN accidentally being transected in different types of gluteal surgeries, close attention was paid to the inferior border of the gluteus maximus muscle. To ensure that branching hooking over the inferior border of the gluteus maximus was not mistaken for pudendal or inferior cluneal nerves, a vertical incision was made in the gluteus maximus muscle following the PFCN course, and the known collateral branches were identified. Data collection was conducted by observing the branching patterns and drawing a detailed representation, as well as taking photographs of each dissection. Each diagram and photo were given a unique donor identification, which was used to distinguish between individuals and compare branching patterns.

## Results

Dissections from a total of 17 specimens resulted in a mean number of 4.59 total PFCN branches per specimen, including an average of 3.47 lateral branches and 0.705 medial branches (Table 1).

**Table 1:** Branching characteristics of posterior femoral cutaneous nerve in 17 specimens

Branching patterns of the PFCN					
Specimen	Medial branches	Lateral branches	Medial curve over G.Max	Lateral curve over G.Max	Total Number of branches
1	0	4	0	1	5
2	0	4	0	1	5
3	0	3	0	0	3
4	0	4	0	1	5
5	5	1	0	0	6
6	0	1	0	0	1
7	1	4	0	0	5
8	0	4	0	0	4
9	0	2	1	1	4
10	0	4	0	1	5
11	0	5	0	0	5
12	2	3	0	0	5
13	3	1	0	0	4
14	0	4	0	0	4
15	1	4	0	0	5
16	0	4	0	1	5
17	0	7	0	0	7

**Figure 1:**
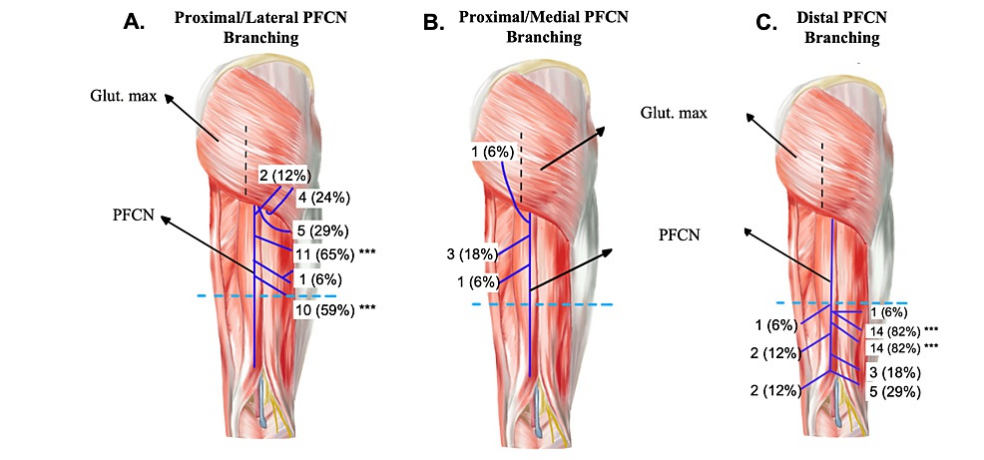
Illustrated branching patterns of PFCN Seven cadavers (41%) had some form of proximal unidentified branching of PFCN over the glut max muscle. (A) Proximal branching was primarily seen coursing laterally in 6 specimens (35%). (B) Only one specimen (6%) had proximal branching of PFCN coursing medially. (C) Distal branching of PFCN was designated by an arbitrary boundary mid-posterior thigh (blue dashed line), roughly halfway through the muscle bellies of semitendinosus, semimembranosus, and biceps femoris muscles. PFCN and its branches are represented by purple lines, which are illustrated to best mimic placement observed on dissection. Branches marked with a ** are commonly illustrated/known branches as seen in current literature. Black dashed lines represent incisions made to follow PFCN deep to gluteus maximus when confirming the origin of unidentified branches.

**Figure 2:**
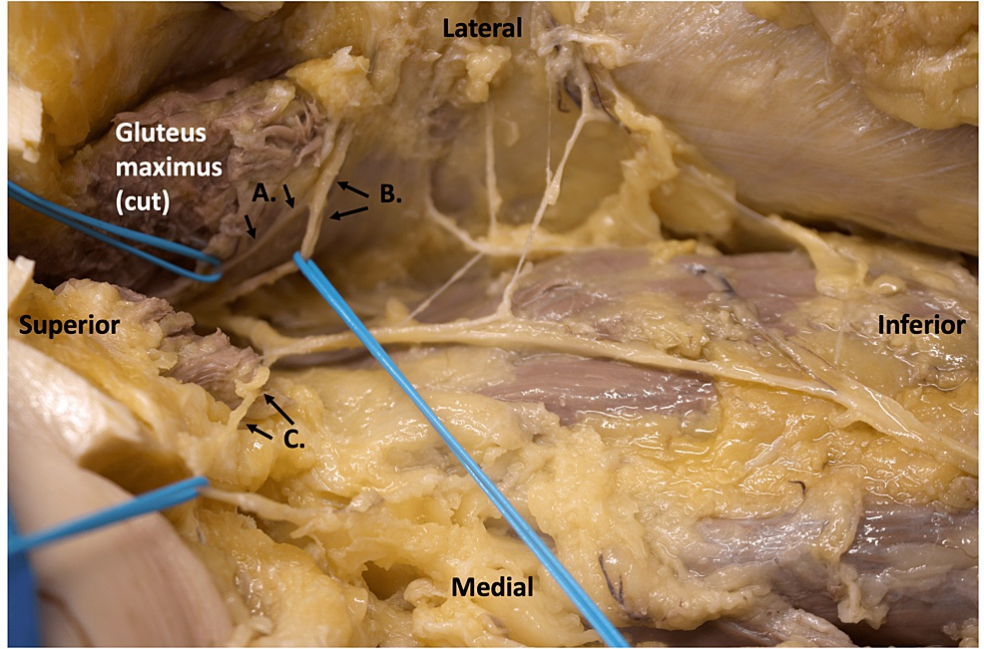
Dissection representation of PFCN branching patterns at the inferior border of gluteus maximus (cut). Gluteus maximus was cut in order to follow the PFCN superiorly and ensure patterns observed were not the known inferior cluneal branches (labeled B). Branching pattern A was seen in 24% of the dissections performed, while branching pattern C was seen in only 6% of cases. Only one case was observed to have both medial and lateral branching of PFCN that hooked over glut max.

Out of 17 specimens, 11 had normal branching of PFCN as commonly seen or illustrated in literature [2,14,15], with no recurrent branches over the inferior edge of the gluteus maximus muscle. Additionally, those 11 specimens (65%) also demonstrated normal PFCN branching with two lateral branches piercing the fascia lata in the proximal posterior thigh (Figure 1). Interestingly, 7 of the 17 specimens (41%) had proximal branching patterns that were recurrent over the inferior border of the gluteus maximus, approximately 3-4 inches from the lateral edge of the muscle (Figure 1A-1B). Out of these 7, only 1 specimen had a proximal branch that coursed medially, while all other specimens (6) had proximal recurrent branches coursing laterally. This recurrent branching pattern was not biased toward sex, as it was observed in three male and three female specimens. Only one specimen had proximal branching of PFCN that was both lateral and medial recurring (Figure 2). This recurrent branching was also not dependent on the side, as this pattern was observed on both the right and left limbs for each sex during this study.

The origin of all proximal recurrent branches was identified through a vertical incision in the gluteus maximus muscle, allowing for visualization of known branches such as inferior cluneal nerves. All recurrent branches observed were confirmed as separate unidentified branches and not known collateral PFCN branches. Other branching patterns observed included five specimens (29%) that had a lateral coursing branch estimated to be 1-2 inches inferior from the border of the gluteus maximus (Figure 1A) and three specimens (18%) that had a medial coursing branch further inferior off PFCN (Figure 1B). These branching patterns did not course over the inferior border of the gluteus maximus but rather pierced the fascia lata to provide cutaneous sensation to the posterior thigh like known PFCN terminal branches.

Distal PFCN branching highly mimicked patterns commonly illustrated in literature [2,14,15], with 14 specimens (82%) having two lateral branches piercing the fascia lata middle-distal posterior thigh just superior to the popliteal fossa (Figure 1C). Lateral branching of PFCN was observed to be the dominant pattern, as only one to two specimens (6-12%) had medial branches in the distal posterior thigh. These results conclude that the current literature accurately depicts the branching patterns of PFCN for its four commonly illustrated terminal branches but does not include the presence of high variability amongst individuals, specifically in the proximal posterior thigh. Knowledge of these recurrent branches is extremely useful as it can help avoid PFCN neuropathy after certain types of surgeries.

## Discussion

The posterior femoral cutaneous nerve is an extensive nerve with numerous collateral branches that provide cutaneous innervation to a large portion of the posterior thigh, infragluteal fold, lateral anal region, scrotum, and labia majora [1-3]. Due to the relationship of the PFCN and its branches to other anatomical structures such as gluteal muscles and ligaments, entrapment may occur in the gluteal region and as it exits to enter the posterior thigh [3,16]. These compression points typically result in different forms of PFCN neuralgia or paresthesia to one or more of its collateral branches [3,17-19]. Multiple studies on PFCN neuropathy conclude that it can either be isolated to a specific collateral branch, a combination of multiple points of entrapment, or even secondary to conditions involving sciatic neuropathy like piriformis syndrome [3,9,20-22]. While these causes of PFCN neuropathy can have non-idiopathic origins such as prolonged cycling, falls in the gluteal region, or sedentary lifestyle and occupations [9,20]; idiopathic or iatrogenic causes of PFCN neuropathy may also occur [3-10]. For example, gluteal surgeries or injections performed to alleviate sciatic neuropathy or pudendal/inferior cluneal neuralgia may risk damaging small unidentified PFCN branches due to injection site, incision placement, or accidental rupture during surgery [3,5-9,16].

Further, it has been noted in multiple studies that patients can experience persistent PFCN neuropathy after surgery for decompression of known collateral branches [3,16]. While this can be due to anatomical variation among individuals, the observations presented in this study provide another explanation for persistent PFCN pain. Unidentified PFCN branches that recurred over the inferior border of the gluteus maximus were observed in 41% of individuals (Figure 1A-1B) and suggest other areas of potential compression or nerve entrapment that could lead to persistent PFCN neuropathy that’s not improved after treatment for sciatic, pudendal, or inferior cluneal neuralgia. Therefore, through this study, we provide insights into delicate unidentified branches of the PFCN to improve well-practiced clinical techniques, post-operative recovery, and patient quality of life.

## Conclusions

In this study, we found that 41% of individuals have an unidentified proximal branch that recurs over the inferior border of the gluteus maximus muscle. Other proximal branching patterns included 29% of individuals with a lateral coursing branch slightly distal to the inferior border of the gluteus maximus and 18% with a medial coursing branch further inferior to PFCN that did not recur. Proximal branching patterns were not side-specific or isolated to a specific sex. Additionally, distal PFCN branching represents commonly illustrated patterns in current literature. In summary, we hope that documentation of unidentified proximal branching patterns of the PFCN allows clinicians to modify current surgical techniques to include other potential sites of entrapment when treating different forms of PFCN neuropathy.
